# State paid family leave policies and breastfeeding duration: cross-sectional analysis of 2021 national immunization survey-child

**DOI:** 10.1186/s13006-024-00646-9

**Published:** 2024-05-26

**Authors:** Julia Rosenberg, Deanna Nardella, Veronika Shabanova

**Affiliations:** 1 Department of Pediatrics, Yale School of Medicine, 333 Cedar St, New Haven, CT, USA; 2 National Clinician Scholars Program, Yale University, 333 Cedar St, New Haven, CT, USA; 3 Department of Biostatistics, Yale School of Public Health, 333 Cedar St, New Haven, CT, USA

**Keywords:** Lactation, Breastfeeding, National Immunization Survey, Family Medical Leave Act

## Abstract

**Background:**

Paid parental leave policies may promote breastfeeding, which can have short- and long-term health benefits for both members of the birthing person-infant dyad. In the United States, where 56% of the workforce qualifies for *unpaid* federal medical leave, certain states have recently enacted *paid* parental and family leave policies. We aimed to assess the extent to which living in states with versus without paid family leave was associated with feeding regimens that included breastfeeding.

**Methods:**

In this cross-sectional analysis of the 2021 National Immunization Survey-Child, we assessed feeding outcomes: (1) exclusively breastfed (only fed breastmilk—never infant formula—both before and after six months of age), (2) late mixed breastfeeding (formula after six months), (3) early mixed breastfeeding (breastfed, formula before six months), and (4) never breastfed. We conducted Pearson χ^2^ to compare social-demographic characteristics and multivariable nominal regression to assess extent to paid family leave was associated with breastfeeding regimens, compared with never breastfeeding.

**Results:**

Of the 35,995 respondents, 5,806 (25% of weighted respondents) were from states with paid family leave policies. Compared with never breastfeeding, all feeding that incorporated breastfeeding—exclusive breastfeeding, late mixed feeding (breastfed, formula introduced after six months), and early mixed feeding (breastfed, formula introduced before six months)—were more prevalent in states with paid family leave policies. The adjusted prevalence ratio (aPR) and differences in adjusted prevalence compared with never breastfeeding in states with versus without paid family leave policies were: aPR 1.41 (95% CI 1.15, 1.73), 5.36% difference for exclusive breastfeeding; aPR 1.25 (95% CI 1.01, 1.53), 3.19% difference for late mixed feeding, aPR 1.32 (95% CI 1.32, 1.97), 5.42% difference for early mixed feeding.

**Conclusion:**

States with paid family leave policies have higher rates of *any* breastfeeding and of *exclusive* breastfeeding than states without such policies. Because all feeding types that incorporate breastfeeding were higher in states with paid family leave policies, expansion of paid family leave may improve breastfeeding rates.

**Supplementary Information:**

The online version contains supplementary material available at 10.1186/s13006-024-00646-9.

## Background

The short- and long-term benefits of breastfeeding for both members of the birthing person-infant dyad have been well-established. The American Academy of Pediatrics, the American College of Obstetrics and Gynecology, and the World Health Organization promote exclusive breastfeeding for six months followed by continued breastfeeding until one to two years of age [1–3]. For newborns, sustained breastfeeding is associated with decreased adverse health consequences including lower rates of infant mortality and lower incidence of sudden infant death syndrome; respiratory, ear, and gastrointestinal infections; asthma; eczema; autoimmune conditions; and diabetes [2]. While exclusive breastfeeding without infant formula introduction is recommended to maximize benefits, data suggest protective effects of *any* breastfeeding against gastrointestinal illnesses, ear infections, asthma, and obesity [4–6]. For birthing people, breastfeeding is associated with decreased rates of hypertension, diabetes, and certain types of cancer [1, 2]. In addition to the individual benefits, breastfeeding promotes dyadic health with opportunities for bonding and benefits for mental and physical health [7]. According to the Centers for Disease Control and Prevention (CDC) breastfeeding report card, in 2019, only 25% of infants born in the United States (US) exclusively breastfed until six months of age and about 36% were breastfeeding at one year [8], falling short of the US Healthy People 2030 targets of 42.4% and 54.1%, respectively [9].

A major barrier to sustained breastfeeding can be caregivers returning to work. Globally, longer maternity leave is associated with higher rates of breastfeeding [10]. In the US, exclusive breastfeeding decreases by approximately 25% within the first week of life, nearly 50% by three months, and 70% by six months, correlating with return to work for many parents [8].

With nearly two-thirds of females participating in the US workforce having a child under three years of age [11], the need to establish and evaluate policies that protect breastfeeding for working lactating persons has been underscored in formal recommendations, including through the Surgeon General’s Calls to Action to Support Breastfeeding and to Improve Maternal Health and the 2022 White House National Strategy on Hunger, Nutrition and Health [12–14]. Despite these recommendations, the US is the only country in the Organization for Economic Cooperation and Development (OECD) to not offer a paid federal family leave policy [15, 16].

**Fig. 1:**
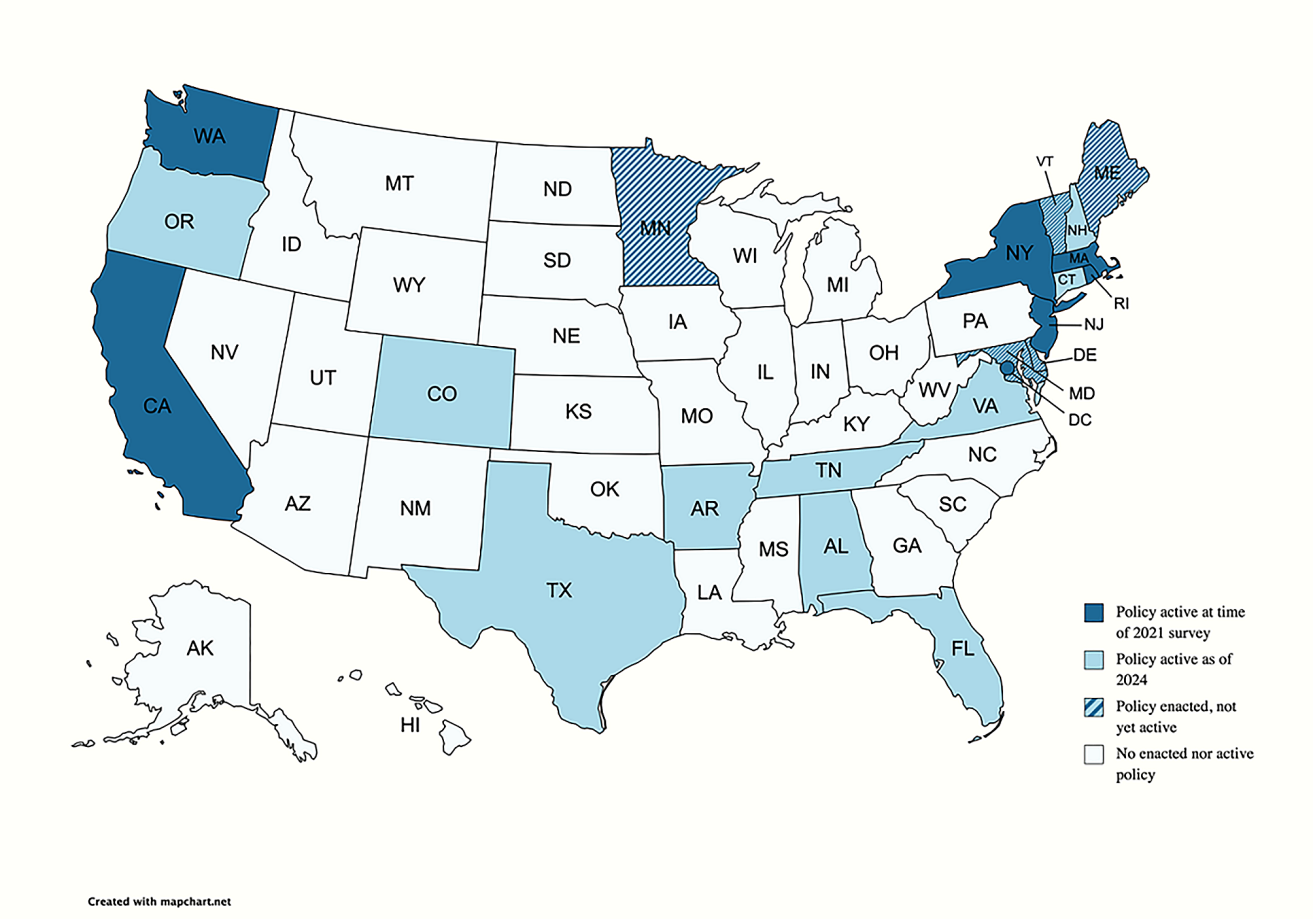
Primary exposure of interest: paid family leave policy status by US state

Federal US Legislation supporting *unpaid* leave and lactation includes the 1993 Family Medical Leave Act (FMLA), the 2010 Patient Protection and Affordable Care Act, and the 2021 Providing Urgent Maternal Protections for Nursing Mothers (PUMP) Act [17]. Federal FMLA stipulates 12 weeks of unpaid, job-protective leave for care of a child [18]. A growing number of states and regions have enacted *paid* family leave policies. As of 2024, 17 US states and Washington, DC have active paid family leave policies, with five states’ legislation to be enacted in future years (Fig. 1) [19].

In this cross-sectional study, we aimed to assess the relationship of paid family leave with a spectrum of breastfeeding outcomes, including metrics of breastfeeding duration and exclusivity, at the population level. We hypothesized that residence in US states with paid family leave policies is associated with higher rates of both exclusive and any breastfeeding.

## Methods

### Data source

This cross-sectional, secondary dataset analysis was conducted using data from the 2021 National Immunization Survey-Child (NIS-C), which included the fifty US states and Washington, DC. The National Immunization Surveys (NIS), which includes NIS-C, are telephone surveys administered via random digit dialing to a stratified representative US sample, conducted by the CDC’s National Center for Immunization and Respiratory Disease. The NIS-C incorporates data from parent/guardian interviews and from questionnaires sent to medical providers to evaluate multiple domains of health, including immunization, breastfeeding, and usage of the Special Supplemental Nutrition Program for Women, Infants, and Children (WIC) program. The 2021 NIS-C target population included US families of children who were 19–35 months old during calendar year 2021. The survey was translated into English and Spanish, and other languages were queried using telephonic interpretation [20, 21].

The NIS-C is a publicly available, de-identified dataset. Its use is not considered Human Subjects Research, and we did not obtain a determination of this status from the Institutional Review Board. We followed the STrengthening the Reporting of OBservational studies in Epidemiology (STROBE) guidelines for cross-sectional observational data (Additional File 1) [22].

### Study variable terminology

When analyzing and presenting data, we used terminology as reported in the NIS-C dataset, which includes terms such as breastfeeding and mother, rather than gender-inclusive terms such as chest feeding and birthing person [23, 24].

### States with and without paid family leave policies

The locations with paid family leave policies at the time of the 2021 NIS-C included California, Massachusetts, New Jersey, New York, Rhode Island, Washington, and Washington, DC (Fig. 1). All other states without active paid family leave policies were grouped together, and Puerto Rico was excluded. Three locations—Washington DC, Massachusetts, and Washington—had aspects of their policies enacted in 2020, so some of the surveyed families may have given birth before full enactment. Thus, *post-hoc* sensitivity analyses were also conducted with these locations removed from analyses.

### Primary outcome

**Fig. 2:**
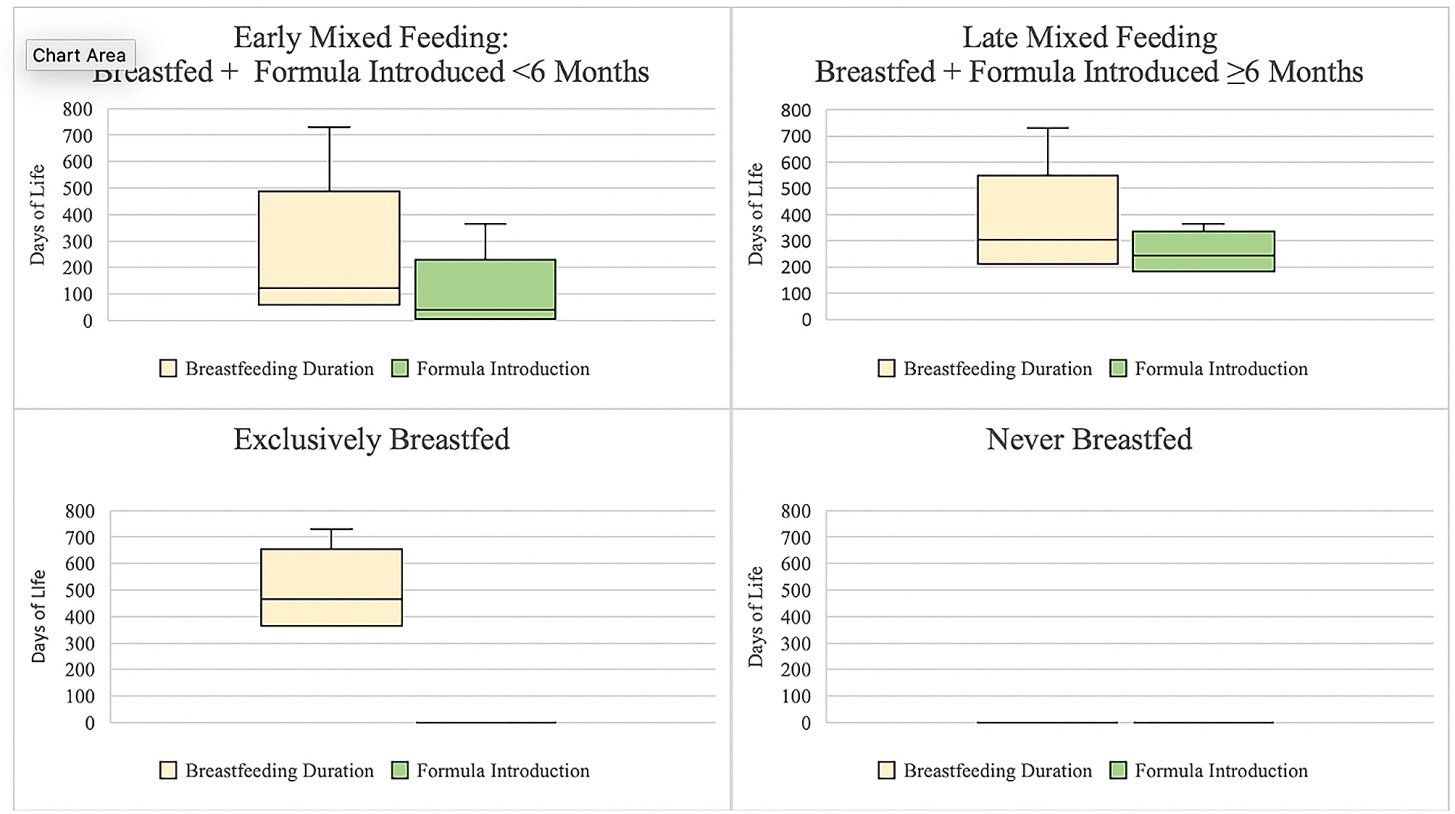
Total days of breastfeeding duration and first day of formula by primary outcome of infant feeding category. Legend: “Exclusively breastfed” refers to receiving breastmilk and never infant formula as source of nutrition but may include supplementary, non-infant formula foods. Figure includes maximum (upper whisker) but not minimum values, as some respondents who reported exclusive breastfeeding had very few days of breastfeeding reported, which we assumed to be errors in reporting. Box indicates upper and lower quartiles. Line indicates median. Adjusted n’s: Exclusively Breastfed *n* = 6,579.82, Late Mixed Feeding *n* = 5,899.27, Early Mixed Feeding *n* = 16,312.40, Never Breastfed *n* = 5,9343.9

The primary outcome was a composite variable reflecting breastmilk and infant formula feeding. To construct this variable, we accounted for three NIS-C variables that assessed if the child ever was fed breastmilk, duration of breastfeeding, and the date of first formula introduction. The finalized infant feeding primary outcome is summarized in Fig. 2 and includes four categories: (1) exclusively breastfed for the duration captured by data (without formula introduction), (2) late mixed breastfeeding (breastfed exclusively until six months, introduced formula after 6 months), and (3) early mixed breastfeeding (breastfed, introduced formula either before six months of age or at unknown time), and (4) never breastfed. “Exclusively breastfed” refers to receiving breastmilk and never infant formula as source of nutrition but may include supplementary, non-infant formula foods. Because the primary outcome centered around breastfeeding, the 141 respondents with unknown breastfeeding data were excluded from the analysis (see Additional File 2 for characteristics of excluded respondents, who could be considered as non-respondents generally due to high rates of missingness for other variables).

### Covariate selection

Based on prior studies of breastfeeding outcomes and available data in the NIS-C, selected covariates included: maternal age, infant age, infant sex, infant race and ethnicity (as reported by the NIS-C dataset), household size, children in the home, maternal education, marital status, language of interviews, poverty level, and WIC enrollment [2]. In order to control for policies that may affect breastfeeding outcomes at the state level, we also developed a covariate to account for workplace lactation protection policies; thirty states and the District of Columbia have such protections in place [25].

### Statistical analyses

Since the data were collected using stratified random sampling, survey responses have been weighted by the weights provided by the NIS-C, to reflect strata and non-response. Simple descriptive statistics (count, percent) and Pearson **χ**^2^ tests were used to describe and compare the social and demographic characteristics of children and mothers from states that did and did not have active paid family leave policies in 2021 (Table 1).

**Table 1:** Sociodemographic characteristics of respondents by residence in states/territories with or without Paid Family Leave Policy, weighted estimates from National Immunization Survey-Child, 2021

Sociodemographic Characteristics	Birthing Person and Infant in States/Territories with Paid Family Leave Policy^a^ % (95% CI)		Birthing Person and Infant in States/Territories without Paid Family Leave Policy ^a^ % (95% CI)	
Infant sex - female	48.84 (46.47, 51.23)		48.91 (47.84, 49.98)	
Has Policy: Breastfeeding in Workplace ***	100 --		56.66 (55.78, 57.53)	
Maternal age ≤ 29 years***	26.60 (24.42, 28.89)		33.03 (31.97, 34.12)	
Race and ethnicity of child***				
Hispanic/Latino	38.42 (35.99, 40.92)		24.17 (23.16, 25.20)	
Non-Hispanic/Non-Latino Black	8.09 (7.03, 9.29)		15.10 (14.21, 16.03)	
Non-Hispanic/Non-Latino other/multiple race	18.64 (16.96, 20.44)		13.22 (12.53, 13.94)	
Non-Hispanic/Non-Latino White	34.85 (32.74, 37.02)		47.51 (46.43, 48.60)	
Infant Age				
19–23 months	30.04 (27.90, 32.27)		30.31 (29.31, 31.32)	
24–29 months	33.78 (31.52, 36.11)		33.60 (32.57, 34.66)	
30–35 months	36.18 (33.98, 38.45)		36.09 (35.07, 37.13)	
Household Size				
2	3.26 (2.51, 4.22)		4.26 (3.81, 4.77)	
3	23.04 (21.18, 25.01)		21.31 (20.45, 22.18)	
4	32.61 (30.47, 34.83)		32.75 (31.71, 33.79)	
≥5	41.09 (38.67, 43.55)		41.69 (40.56, 42.82)	
Children in Home*				
1	30.11 (27.99, 32.31)		27.33 (26.37, 28.32)	
2–3	56.85 (54.44, 59.23)		57.56 (56.44, 58.67)	
≥4	13.04 (11.34, 14.96)		15.10 (14.27, 15.98)	
Highest Education of Birthing Person***				
<12 years	10.29 (8.61, 12.26)		9.16 (8.49, 9.88)	
≥12 years, non-college graduate	45.02 (42.62, 47.45)		52.13 (51.02, 53.24)	
College graduate	44.69 (42.37, 47.03)		38.71 (37.67, 39.75)	
Birthing Person Marital Status***				
Married	66.39 (63.96, 68.74)		61.21 (60.07, 62.33)	
Other Marital Status	33.61 (31.26, 36.04)		38.79 (37.67, 39.93)	
Language of Interview***				
English	87.87 (85.92, 89.57)		93.97 (93.39, 94.50)	
Spanish	9.02 (7.49, 10.82)		5.35 (4.83, 5.91)	
Other	3.12 (2.35, 4.13)		0.68 (0.55, 0.85)	
Poverty Level, Family Income***				
Below poverty level	21.31 (19.23, 23.55)		24.13 (23.08, 25.21)	
Above poverty level, ≤$75,000	27.61 (25.45, 29.89)		31.57 (30.55, 32.61)	
Above poverty level, >$75,000	42.35 (40.06, 44.67)		38.07 (37.02, 39.13)	
Unknown	8.72 (7.37, 10.30)		6.23 (5.70, 6.82)	
WIC Enrollment**				
Enrolled	40.99 (38.58, 43.45)		44.94 (43.81, 46.07)	
Not enrolled	57.86 (55.41, 60.28)		54.44 (53.31, 55.57)	
Unknown or refused to answer	1.15 (0.74, 1.76)		0.62 (0.46, 0.84)	

^a^Six states and Washington, DC with paid family leave policies (as of 2021): California, Massachusetts, New Jersey, New York, Rhode Island, Washington, Washington DC. Percentages and 95% confidence intervals (CI) are survey weighted. For birthing person and infant in state/territory with paid leave policy, observed *n* = 5,806 and weighted *n* = 4,060,975. For birthing person and infant in state/territory without paid leave policy, observed *n* = 28,916 and weighted *n* = 1,362,335. Pearson χ^2^: *p < 0.05; **p < 0.01; ***p < 0.001.

We used multivariable nominal regression to examine the extent to which the primary exposure variable of residence in states with and without paid family leave policies in 2021 was associated with the primary outcome of any infant feeding regimen that included breastfeeding, compared with never breastfeeding. In this multivariable regression, we controlled for *a priori* chosen covariates, all of which were retained in the adjusted model and are displayed in Tables 1 and 2. Results are reported as adjusted estimates of prevalence of each feeding regimen (as percentages within each outcome level); differences between adjusted prevalence of breastfeeding outcomes (exclusive, late mixed, early mixed) and adjusted prevalence of never breastfeed; and unadjusted and adjusted prevalence ratios (PR, aPR) with 95% confidence intervals (CI), which were obtained postestimation via linear combinations of relevant parameters and use of marginal mean statement [26]. As noted in the description of the independent variable (states with and without family leave policies), we also conducted *post-hoc* sensitivity analyses without states where implementation of policies occurred during the study period. Because we were interested in the effect of paid family leave on a spectrum of breastfeeding outcomes that corresponded to independent hypotheses, we did not adjust for multiple comparisons [27, 28].

**Table 2:** Unadjusted and adjusted prevalence ratio of mixed and exclusive breastfeeding outcomes compared with never breastfed, weighted estimates from National Immunization Survey-Child, 2021

	Exclusively Breastfed versus Never Breastfed PR (95% CI)		Late Mixed Feeding^a^ versus Never Breastfed PR (95% CI)		Early Mixed Feeding^b^ versus Never Breastfed PR (95% CI)	
	Unadjusted	Adjusted	Unadjusted	Adjusted	Unadjusted	Adjusted
Resides in State with Paid Family Leave Policy	1.72 (1.43, 2.08)	1.41 (1.15, 1.73)	1.61 (1.32, 1.97)	1.25 (1.01, 1.53)	1.61 (1.35, 1.91)	1.32 (1.10, 1.58)
Has State Policy: Breastfeeding in Workplace	1.59 (1.37, 1.85)	1.36 (1.16, 1.60)	1.53 (1.30, 1.79)	1.28 (1.08, 1.51)	1.45 (1.27, 1.66)	1.24 (1.07, 1.43)
Infant sex - female	0.99 (0.89, 1.18)	1.03 (0.89, 1.19)	1.02 (0.89, 1.18)	1.05 (0.91, 1.21)	0.89 (0.79, 1.00)	0.91 (0.80, 1.03)
Maternal age ≤ 29 years	0.52 (0.45, 0.61)	1.25 (1.05, 1.50)	0.63 (0.54, 0.74)	1.14 (0.96, 1.37)	0.76 (0.67, 0.87)	1.10 (0.95, 1.27)
Race/ethnicity of child						
Hispanic/Latino	0.54 (0.45, 0.65)	0.84 (0.67, 1.04)	0.96 (0.80, 1.16)	1.25 (1.01, 1.54)	0.95 (0.81, 1.12)	1.17 (0.97, 1.41)
Non-Hispanic/Non-Latino Black	0.25 (0.20, 0.31)	0.54 (0.42, 0.69)	0.58 (0.47, 0.72)	1.09 (0.87, 1.37)	0.60 (0.50, 0.72)	0.89 (0.74, 1.08)
Non-Hispanic/Non-Latino other/multiple race	0.80 (0.65, 0.98)	0.93 (0.75, 1.15)	1.13 (0.92, 1.41)	1.24 (0.99, 1.54)	1.07 (0.90, 1.29)	1.13 (0.94, 1.36)
Non-Hispanic/Non-Latino White	Reference	Reference	Reference	Reference	Reference	Reference
Infant Age						
19–23 months	1.12 (0.95, 1.32)	1.09 (0.92, 1.30)	1.00 (0.84, 1.20)	0.99 (0.83, 1.19)	1.23 (1.06, 1.42)	1.20 (1.04, 1.40)
24–29 months	1.10 (0.93, 1.29)	1.06 (0.89, 1.26)	1.06 (0.89, 1.26)	1.03 (0.87, 1.23)	1.16 (1.00, 1.35)	1.13 (0.98, 1.32)
30–35 months	Reference	Reference	Reference	Reference	Reference	Reference
Household Size						
2	0.43 (0.30, 0.63)	1.11 (0.66, 1.86)	0.65 (0.44, 0.96)	0.90 (0.55, 1.47)	1.10 (0.82, 1.47)	1.27 (0.89, 1.83)
3	1.27 (0.97, 1.41)	1.10 (0.80, 1.52)	1.22 (1.00, 1.48)	0.89 (0.67, 1.19)	1.70 (1.44, 2.01)	1.29 (1.03, 1.62)
4	1.27 (1.07, 1.51)	0.89 (0.74, 1.08)	1.27 (1.07, 1.51)	0.94 (0.77, 1.15)	1.41 (1.21, 1.64)	1.11 (0.94, 1.32)
≥5	Reference	Reference	Reference	Reference	Reference	Reference
Children in Home						
1	1.07 (0.86, 1.34)	0.59 (0.41, 0.86)	1.43 (1.12, 1.83)	1.14 (0.81, 1.62)	1.94 (1.59, 2.37)	1.27 (0.96, 1.67)
2–3	1.32 (1.08, 1.61)	0.82 (0.64, 1.04)	1.42 (1.14, 1.77)	1.02 (0.80, 1.31)	1.56 (1.29, 1.88)	1.14 (0.92, 1.40)
≥4	Reference	Reference	Reference	Reference	Reference	Reference
Highest Education of Birthing Person						
<12 years	0.13 (0.09, 0.18)	0.28 (0.19, 0.41)	0.26 (0.20, 0.35)	0.40 (0.28, 0.56)	0.30 (0.24, 0.37)	0.41 (0.31, 0.54)
≥12 years, non-college graduate	0.21 (0.18, 0.25)	0.42 (0.34, 0.50)	0.31 (0.26, 0.37)	0.53 (0.44, 0.65)	0.40 (0.35, 0.47)	0.58 (0.49, 0.69)
College graduate	Reference	Reference	Reference	Reference	Reference	Reference
Birthing Person Marital Status						
Married	4.40 (3.76, 5.16)	1.89 (1.55, 2.30)	2.81 (2.40, 3.28)	1.58 (1.31, 1.92)	2.10 (1.84, 2.38)	1.45 (1.24, 1.70)
Other Marital Status	Reference	Reference	Reference	Reference	Reference	Reference
Language of Interview						
English	Reference	Reference	Reference	Reference	Reference	Reference
Spanish	0.75 (0.52, 1.07)	2.00 (1.30, 3.09)	1.48 (1.07, 2.03)	2.70 (1.83, 3.96)	1.15 (0.88, 1.50)	1.71 (1.24, 2.37)
Other	0.34 (0.18, 0.64)	0.46 (0.23, 0.91)	1.13 (0.63, 2.04)	1.35 (0.73, 2.52)	1.01 (0.61, 1.68)	1.12 (0.64, 1.96)
Poverty Level, Family Income						
Below poverty level	0.19 (0.15, 0.23)	0.93 (0.71, 1.21)	0.28 (0.23, 0.34)	0.68 (0.51, 0.89)	0.42 (0.35, 0.49)	0.93 (0.75, 1.15)
Above poverty level, ≤$75,000	0.43 (0.36, 0.51)	1.24 (1.02, 1.51)	0.45 (0.38, 0.54)	0.86 (0.70, 1.07)	0.61 (0.52, 0.72)	1.00 (0.84, 1.20)
Above poverty level, >$75,000	Reference	Reference	Reference	Reference	Reference	Reference
Unknown	0.35 (0.26, 0.48)	0.92 (0.66, 1.29)	0.52 (0.38, 0.71)	0.88 (0.63, 1.22)	0.53 (0.41, 0.69)	0.87 (0.66, 1.15)
WIC Enrollment						
Enrolled	0.17 (0.14, 0.20)	0.32 (0.26, 0.39)	0.30 (0.26, 0.35)	0.49 (0.40, 0.61)	0.44 (0.39, 0.50)	0.69 (0.58, 0.82)
Not enrolled	Reference	Reference	Reference	Reference	Reference	Reference
Unknown or refused to answer	0.38 (0.14, 1.04)	0.52 (0.16, 1.68)	0.51 (0.23, 1.14)	0.62 (0.26, 1.47)	0.54 (0.27, 1.10)	0.63 (0.30, 1.32)

^a^Formula introduce after 6 completed months or later. ^b^Formula introduced before 6 months or at unknown time. PR: prevalence ratio. CI: confidence interval. WIC: Women, Infants and Children. Adjusted analyses includes all listed variables.

Given the predetermined sample size by the NIS-C, our conclusions are based on the magnitude of the PR and surrounding 95% CI, rather than the p-values [29, 30]. We further defined a meaningful magnitude of difference in the prevalence of breastfeeding at the 1% point, which reflects the change noted in the prior three years of exclusive breastfeeding in the United States, per Healthy People 2030 [9].

We completed analyses in Stata Version 15 (StataCorp College Station, Texas).

## Results

### Sociodemographic characteristics by state paid family leave policy

As shown in Table 1, of the 34,722 NIS-C unweighted survey respondents from 2021, 5,806 were from states with paid family leave policies, and they accounted for 25% of weighted survey respondents. We did not observe a meaningful difference between states with and without paid family leave policies in terms of infant sex, infant age, or household size. Differences were noted when assessing breastfeeding workplace policies, maternal age, number of children in the home, parental education, marital status, language of the interview, poverty level, and WIC enrollment (Table 1). Families in states with paid parental leave policies were more likely to reside in states that had workplace breastfeeding protections, report that the mother was over 29 years old, the infant was of Hispanic/Latino ethnicity, there were fewer children in the home, the mother graduated college, the mother was married, and that the income was above poverty and above $75,000. They were less likely to report being enrolled in WIC (Table 1).

### Infant feeding patterns by state paid family leave policy

**Fig. 3:**
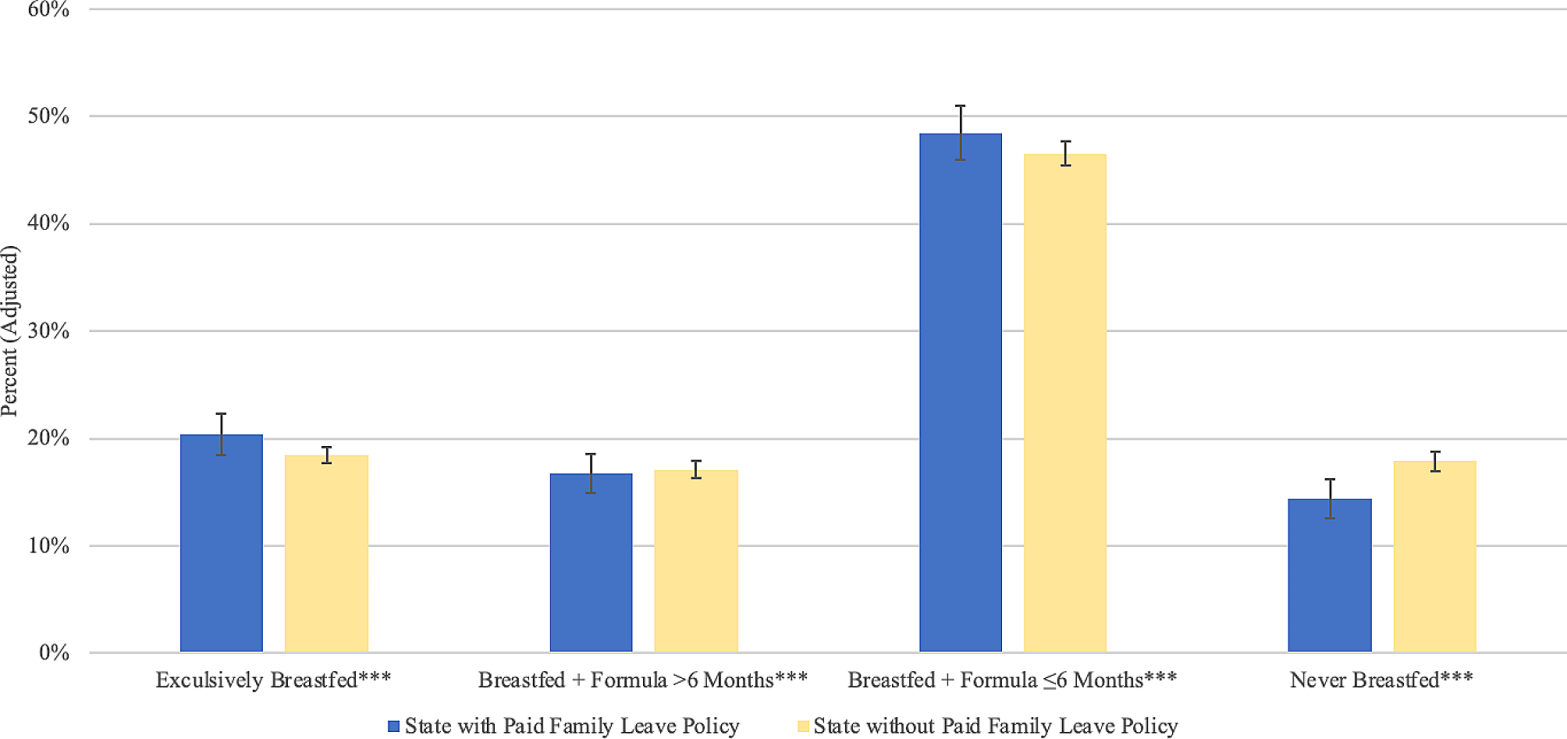
Adjusted prevalence of breastfeeding outcomes by US state paid family leave policies, National Immunization Survey-Child, 2021. Legend: Reported values are estimated prevalence of each feeding type. Error bars correspond to 95% confidence intervals

Exclusive breastfeeding was more prevalent in states with paid family leave policies than in states without such policies: 20.36% (95% CI 18.46%, 22.26%) in states with policies vs. 18.48% (95% CI 17.74%, 19.22%) in states without policies. Late mixed breastfeeding was similar in states with vs. without paid family leave: 16.78% (95% CI 14.97%, 18.60%) vs. 17.09% (95% CI 16.30%, 17.88%). Early mixed breastfeeding was more prevalent in states with vs. without paid family leave: 48.48% (95% CI 46.00%, 50.97%) vs. 46.55% (95% CI 45.47%, 47.64%). Fewer reported never breastfeeding in states with paid family leave: 14.38% (95% CI 12.52%, 16.23%) vs. 17.87% (95% CI 16.98%, 18.76%) (Fig. 3).

**Fig. 4:**
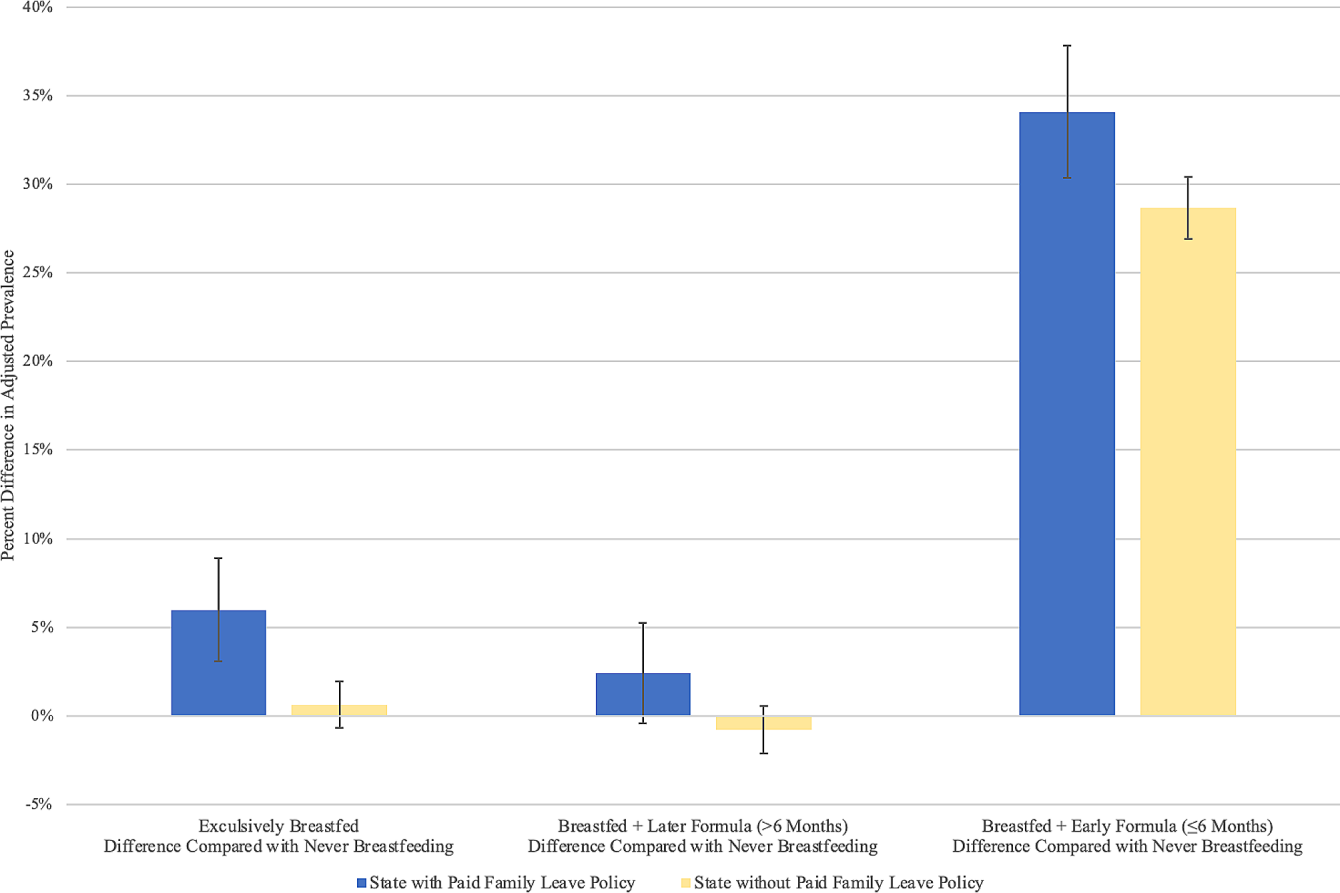
Difference in adjusted prevalence of breastfeeding outcomes and prevalence of never breastfeeding by US state paid family leave policies, National Immunization Survey-Child, 2021. Legend: Reported values are estimated differences in adjusted prevalence of each feeding type. Error bars correspond to 95% confidence intervals

As shown in Table 2; Fig. 4, compared with never breastfeeding, the adjusted prevalence of exclusive breastfeeding was higher by 5.36% (aPR = 1.41) in states with paid family leave vs. in states without paid family leave. Similarly, the adjusted prevalence of late mixed breastfeeding was higher by 3.19% (aPR = 1.25), and early mixed breastfeeding was higher by 5.42% (aPR = 1.32).

A sensitivity analysis which excluded states that enacted family leave policies during the study period (Washington DC, Washington, and Massachusetts) found similar results across all feeding types (Additional File 3).

### Covariates associated with breastfeeding

As shown in Table 2, several covariates remained associated with breastfeeding outcomes in the adjusted model.

Workplace breastfeeding policies were positively associated with all types of breastfeeding.

Women with income below poverty had lower aPR of late mixed breastfeeding. Women who were married had greater aPR of all breastfeeding outcomes. Respondents who reported the race/ethnicity of their child as non-Hispanic/Latino Black had lower aPR of exclusively breastfeeding compared with non-Hispanic/Latino White children. Respondents who identified their children as Hispanic/Latino had higher aPR of late mixed feeding compared with non-Hispanic/Latino White children. Compared with English-speaking respondents, Spanish-speaking respondents had higher aPR of all breastfeeding types. Respondents with lower education levels had lower aPR of all breastfeeding outcomes. WIC enrollees also had lower aPR of all types of breastfeeding.

## Discussion

In this secondary dataset analysis using a US representative sample, we found that, after adjusting for covariates, all feeding types that incorporate breastfeeding (exclusive breastfeeding, late mixed feeding, and/or early mixed feeding) were higher in states with paid family leave compared with states without paid family leave policies. These findings offer novel insight into various feeding approaches indicative of real-world infant feeding practices among breastfeeding persons in the labor force. Even after controlling for state-level workplace breastfeeding policies, paid family leave policies are associated with exclusive breastfeeding and with a spectrum of feeding types that incorporate breastfeeding. These results demonstrate that the paid family leave policies are positively associated with breastfeeding, and thus may reflect health benefits for the parent/baby dyad that accompany *any* degree of breastfeeding [2, 4–6].

Paid family leave policies have previously been associated with improvements in breastfeeding duration and with health and economic benefits [31–36]. After California was the first US state to provide eight weeks of partial paid family leave in 2004, Huang et al. utilized the CDC Infant Feeding Practices Study and found contemporaneous increases in the rates of breastfeeding in California compared with other US states, with difference-in-differences of 15.8%, 17.4%, and 18.4% in the rates of any breastfeeding at three, six, and nine months, respectively [37, 38]. Globally, parental leave after childbirth has been associated with reduced maternal and infant morbidity and mortality [15, 39–42]. In high-income countries, paid parental leave has been associated with increases in exclusive breastfeeding, downstream earning potential, workforce retention, and infant vaccination rates and with reductions in maternal medical and mental health morbidity [16, 43]. Data suggest higher degrees of benefit with increasingly generous leave, including longer breastfeeding duration and higher maternal pay [44].

Just over half (56%) of the US workforce qualifies for federal FMLA, which is unpaid [45]. There are also limited opportunities for paid leave in the United States, which results in suboptimal breastfeeding initiation and duration [41]. Cross-sectional US studies have found that 59% of women did not receive paid leave, and, even when it was received, paid leave averaged about three weeks, with reduced salary [46]. Current FMLA policies and qualifications tend to support families who can afford unpaid time off work and have stable employment from large employers [18]. Women facing social and structural barriers to breastfeeding, such as low income, lower educational attainment, and membership in minority racial and ethnic groups, disproportionately do not benefit from federal FMLA policies. In this multivariable analysis, we found disparities in breastfeeding was associated with multiple factors that are also related to disparities in medical leave policies.

When evaluating income, we found that, in the adjusted model, women below the poverty level were less likely to exclusively breastfeed for the first six months before introducing infant formula. Families with lower incomes have previously been shown to have less paid and unpaid leave. One 2014 cross-sectional study found that only 20% of families making under $35,000 per year received paid leave, averaging 1.5 weeks in duration, compared with a respective 55% and 4.5 weeks for families making over $75,000 per year [46]. The 2018 US Department of Labor FMLA surveys demonstrated that low-wage workers making $15 per hour or less were least likely to take needed medical leave, citing the inability to afford unpaid time off from work and fear of job loss [45].

Similar to other studies, we found that marital status was positively associated with breastfeeding [47]. The US Department of Labor reports that approximately 95% of fathers with children under three years of age are working, highlighting the potential importance of parental leave for all caregivers in the workforce [11].

Our findings also reflected known racial and ethnic disparities in breastfeeding stemming from complex policies and histories related to structural and ongoing racism [8, 48, 49]. After adjusting for covariates, we found that, compared with respondents who identified their children as non-Hispanic White, non-Hispanic Black respondents were less likely to exclusively breastfeed. Racial and ethnic disparities extend to FMLA eligibility; studies have found that more Black and Hispanic/Latino workforce members (60.2% and 66.9%, respectively) reported being ineligible for or unable to afford unpaid leave than White workers (55.3%) [39]. Because Black women experience higher rates of pregnancy complications and preterm delivery compared to other races, current federal FMLA policies, which count time from pregnancy complications as part of leave, may further exacerbate racial inequities [50–52]. We also found that Hispanic/Latino respondents had higher rates of late mixed feeding but lower rates of exclusive breastfeeding compared with non-Hispanic White women. A 2021 study examining feeding goals found that despite Hispanic/Latina women having higher intentions to breastfeeding compared with non-Hispanic/Latina White women, they had lower odds of meeting their goals [53]. Inequitable access to FMLA policies may be contributing to this gap, as Hispanic/Latina women are less likely to qualify for both paid and unpaid leave, which may be related to part-time work status or working for small employers [39, 54, 55].

Employment and education have also previously been associated with breastfeeding outcomes and may be related to access to FMLA [47]. While NIS-C does not include employment data, in the unadjusted and adjusted analyses, we found that women with lower education levels, which are associated with employment opportunities, were less likely to breastfeed. Return to work is among the top reasons for interrupted breastfeeding [33, 56], and women who take six months or more of leave from work have a 30% higher likelihood of any breastfeeding at six months [57].

We also found that women enrolled in WIC were less likely to report breastfeeding across all adjusted and unadjusted analyses. For breastfeeding individuals, WIC distributes breast pumps, offers nutritional support, and supports breastfeeding peer counselor programs [58]. WIC also subsidizes formula purchases, which can result in sales benefits for formula manufactures [59]. Multi-level strategies have been found to enhance breastfeeding for WIC participants, including supporting early WIC enrollment, assessing breastfeeding intentions, and funding peer counseling [60]. However, formula provision may be an incentive for WIC enrollment for some income-eligible individuals, and prior studies have found that some enrollees perceive WIC as a formula provider and appreciate the financial support for formula supplementation [61]. These findings underscore the need to further enrich the lactation-supporting capacity of WIC while considering financial implications and regulations for formula provision.

Limitations of this cross-sectional, secondary dataset analysis included baseline differences between the states with and without paid family leave policies. Respondents in states with paid family leave policies were more likely to report older age, Hispanic/Latina ethnicity, smaller household size, college degree, married status, higher income levels, and lower WIC enrollment. While we adjusted for these characteristics and for workplace breastfeeding policies, there were potentially other unmeasured confounding factors that may have differed between states with and without paid family leave policies, including maternal employment status. Although more generous leave has been found to be associated with greater benefits for breastfeeding-related outcomes, this study did not account for state-by-state variation in leave policies [44]. Several states had policies that went into effect during the survey lookback period, but sensitivity analyses showed similar outcomes regardless of inclusion or exclusion of these states in analyses. Additionally, there were limited data for families who prefer languages other than English or Spanish, and birthing people with varying gender identities may have been excluded.

Despite the limitations of the cross-sectional analysis, it is important to assess differences in breastfeeding outcomes, including mixed breast and formula feeding, as more states enact paid family leave policies. Future prospective studies can evaluate changes in breastfeeding after policy enactment and can assess breastfeeding prevalence in the workforce.

## Conclusions

In the United States, all feeding types that incorporate breastfeeding were higher in states with paid family leave compared with states without paid family leave policies. Although multilevel interventions are needed to support breastfeeding, expansion of policies that grant working families and caregivers paid time to raise children can positively affect breastfeeding, which in turn could improve preventative health and economic benefits for individuals and society.

### Electronic supplementary material

Below is the link to the electronic supplementary material.

Supplementary Material 1

Supplementary Material 2

Supplementary Material 3

## Data Availability

The datasets analyzed during the current study are available in the repository which is available from the Centers for Disease Control and Prevention at: https://www.cdc.gov/vaccines/imz-managers/nis/datasets.html.

## Abbreviations

aPR: Adjusted prevalence ratio
CDC: Centers for Disease Control and Prevention
CI: Confidence Interval
FMLA: Family Medical Leave Act
NIS: National Immunization Surveys
NIS-C: National Immunization Survey-Child
OECD: Organization for Economic Cooperation and Development
PR: Prevalence Ratio
PUMP: Providing Urgent Maternal Protections for Nursing Mothers Act
STROBE: STrengthening the Reporting of OBservational studies in Epidemiology
US: United States
WIC: Special Supplemental Nutrition Program for Women, Infants, and Children
