# Supplementary material for: State paid family leave policies and breastfeeding duration: cross-sectional analysis of 2021 national immunization survey-child
Source: Int Breastfeed J. 2024 May 26;19:37. doi: 10.1186/s13006-024-00646-9 (PMC11128124; doi:10.1186/s13006-024-00646-9)
Supplement: Supplementary file 2 — Supplementary Material 2 [file 13006_2024_646_MOESM2_ESM.docx]

**Additional File 2. Sociodemographic characteristics of nonrespondents, National Immunization Survey-Child, 2021**

| **Sociodemographic characteristics of nonrespondents, National Immunization Survey-Child, 2021** | | | | |
| --- | --- | --- | --- | --- |
| **Sociodemographic**  **Characteristics** | **Excluded, Missing Breastfeeding Data (observed n = 141)**  **% (95% CI)** | | **Included, Has Breastfeeding Data (observed n = 34.722)**  **% (95% CI)** | |
| Has State Policy: Breastfeeding in Workplace* | 48.71 | (30.92, 66.84) | 67.55 | (66.77, 68.31) |
| Infant sex - female | 43.71 | (32.82, 55.25) | 48.89 | (47.90, 49.89) |
| Maternal age ≤29 years | 47.06 | (28.69, 66.25) | 31.42 | (30.45, 32.40) |
| Race/ethnicity of child |  |  |  |  |
| Hispanic/Latino | 22.69 | (12.05, 38.61) | 27.75 | (26.76, 28.76) |
| Non-Hispanic/Non-Latino Black | 17.22 | (8.73, 31.13) | 13.34 | (12.61, 14.10) |
| Non-Hispanic/Non-Latino other/multiple race | 14.85 | (7.43, 27.48) | 14.58 | (13.91, 15.28) |
| Non-Hispanic/Non-Latino White | 45.24 | (26.99, 64.87) | 44.33 | (43.35, 45.31) |
| Infant Age |  |  |  |  |
| 19-23 months | 21.84 | (11.73, 37.02) | 30.24 | (29.32, 31.18) |
| 24-29 months | 45.29 | (27.37, 64.52) | 33.65 | (32.68, 34.63) |
| 30-35 months | 32.87 | (20.71, 47.85) | 36.12 | (35.17, 37.08) |
| Household Size |  |  |  |  |
| 2 | 0.84 | (0.26, 2.67) | 4.01 | (3.62, 4.45) |
| 3 | 11.81 | (4.98, 25.51) | 21.74 | (20.95, 22.56) |
| 4 | 47.27 | (28.94, 66.37) | 32.71 | (31.77, 33.67) |
| ≥5 | 40.08 | (25.09, 57.18) | 41.53 | (40.50, 42.48) |
| Children in Home |  |  |  |  |
| 1 | 16.75 | (8.29, 30.92) | 28.03 | (27.13, 28.95) |
| 2-3 | 58.29 | (40.86, 73.87) | 57.38 | (56.35, 58.41) |
| ≥4 | 24.96 | (14.76, 39.00) | 14.59 | (13.82 15.39) |
| Highest Education of Birthing Person |  |  |  |  |
| <12 years | 13.12 | (6.24, 25.52) | 9.45 | (8.78, 10.16) |
| ≥12 years, non-college graduate | 55.60 | (37.78, 72.08) | 50.34 | (49.31, 51.38) |
| College graduate | 31.29 | (18.59, 47.49) | 40.21 | (39.24, 41.19) |
| Birthing Person Marital Status* |  |  |  |  |
| Married | 56.00 | (38.14, 72.43) | 62.51 | (61.47, 63.54) |
| Other Marital Status | 44.00 | (27.57, 61.86) | 37.49 | (36.46, 38.53) |
| Language of Interview |  |  |  |  |
| English | 97.76 | (89.72, 99.54) | 92.43 | (91.79, 93.04) |
| Spanish | 2.24 | (0.46, 10.28) | 6.27 | (5.71, 6.88) |
| Other | 0 | -- | 1.30 | (1.07, 1.57) |
| Poverty Level, Family Income** |  |  |  |  |
| Below poverty level | 18.72 | (9.38, 33.88) | 23.42 | (22.47, 24.40) |
| Above poverty level, ≤$75,000 | 22.62 | (11.70, 39.21) | 30.58 | (29.64, 31.53) |
| Above poverty level, >$75,000 | 32.69 | (19.80, 48.86) | 39.14 | (38.17, 40.13) |
| Unknown | 25.96 | (9.60, 53.67) | 6.86 | (6.32, 7.44) |
| WIC Enrollment*** |  |  |  |  |
| Enrolled | 46.41 | (29.32, 64.39) | 43.95 | (42.91, 44.99) |
| Not enrolled | 20.84 | (11.52, 34.47) | 55.30 | (54.25, 56.34) |
| Unknown or refused to answer | 32.75 | (14.90, 57.53) | 0.75 | (0.59, 0.97) |
| Percentages and 95% confidence intervals (CI) are survey weighted. For excluded cohort with missing breastfeeding data, observed n = 144 and weighted n = 26,872. For birthing person and infant in state/territory without paid leave policy, observed n = 35,851 and weighted n = 5,423,312. Pearson χ^2^: *p <0.05; **p <0.01; ***p <0.001. | | | | |
