# Supplementary material for: State paid family leave policies and breastfeeding duration: cross-sectional analysis of 2021 national immunization survey-child
Source: Int Breastfeed J. 2024 May 26;19:37. doi: 10.1186/s13006-024-00646-9 (PMC11128124; doi:10.1186/s13006-024-00646-9)
Supplement: Supplementary file 3 — Supplementary Material 3 [file 13006_2024_646_MOESM3_ESM.docx]

**Additional File 3: Sensitivity Analysis without Washington, DC, Massachusetts, or Washington**

| **Sensitivity Analysis without Washington, DC, Massachusetts, or Washington.** Unadjusted and Adjusted Prevalence Ratio of Mixed and Exclusive Breastfeeding Outcomes Compared with Never Breastfed, Weighted Estimates from National Immunization Survey-Child, 2021 | | | | | | |
| --- | --- | --- | --- | --- | --- | --- |
|  | Early Mixed Feeding^a^ versus  Never Breastfed  PR (95% CI) | | Late Mixed Feeding^b^ versus  Never Breastfed  PR (95% CI) | | Exclusively Breastfed versus  Never Breastfed  PR (95% CI) | |
|  | Unadjusted | Adjusted | Unadjusted | Adjusted | Unadjusted | Adjusted |
| Resides in State with Paid Family Leave Policy | 1.59 (1.32, 1.93) | 1.30 (1.06, 1.59) | 1.58 (1.26, 1.98) | 1.21 (0.96, 1.53) | 1.62 (1.31, 2.01) | 1.33 (1.06, 1.68) |
| Has State Policy: Breastfeeding in Workplace | 1.45 (1.27, 1.66) | 1.24 (1.07, 1.43) | 1.53 (1.30, 1.79) | 1.27 (1.08, 1.51) | 1.59 (1.37, 1.85) | 1.36 (1.16, 1.60) |
| Infant sex - female | 0.89 (0.79, 1.00) | 0.91 (0.80, 1.03) | 1.02 (0.89, 1.18) | 1.06 (0.91, 1.23) | 0.99 (0.89, 1.18) | 1.02 (0.88, 1.19) |
| Maternal age ≤29 years | 0.76 (0.67, 0.87) | 1.10 (0.95, 1.28) | 0.63 (0.54, 0.74) | 1.15 (0.96, 1.38) | 0.52 (0.45, 0.61) | 1.24 (1.04, 1.49) |
| Race/ethnicity of child |  |  |  |  |  |  |
| Hispanic/Latino | 0.95 (0.81, 1.12) | 1.16 (0.96, 1.41) | 0.96 (0.80, 1.16) | 1.27 (1.02, 1.58) | 0.54 (0.45, 0.65) | 0.84 (0.68, 1.05) |
| Non-Hispanic/Non-Latino Black | 0.60 (0.50, 0.72) | 0.89 (0.73, 1.08) | 0.58 (0.47, 0.72) | 1.09 (0.86, 1.38) | 0.25 (0.20, 0.31) | 0.55 (0.42, 0.70) |
| Non-Hispanic/Non-Latino other/multiple race | 1.07 (0.90, 1.29) | 1.14 (0.94, 1.39) | 1.13 (0.92, 1.41) | 1.26 (1.01, 1.59) | 0.80 (0.65, 0.98) | 0.97 (0.78, 1.21) |
| Non-Hispanic/Non-Latino White | Reference | Reference | Reference | Reference | Reference | Reference |
| Infant Age |  |  |  |  |  |  |
| 19-23 months | 1.23 (1.06, 1.42) | 1.21 (1.03, 1.41) | 1.00 (0.84, 1.20) | 0.99 (0.82, 1.20) | 1.12 (0.95, 1.32) | 1.11 (0.93, 1.32) |
| 24-29 months | 1.16 (1.00, 1.35) | 1.14 (0.98, 1.33) | 1.06 (0.89, 1.26) | 1.02 (0.85, 1.22) | 1.10 (0.93, 1.29) | 1.06 (0.89, 1.27) |
| 30-35 months | Reference | Reference | Reference | Reference | Reference | Reference |
| Household Size |  |  |  |  |  |  |
| 2 | 1.10 (0.82, 1.47) | 1.29 (0.90, 1.90) | 0.65 (0.44, 0.96) | 0.92 (0.56, 1.52) | 0.43 (0.30, 0.63) | 1.14 (0.67, 1.94) |
| 3 | 1.70 (1.44, 2.01) | 1.34 (1.06, 1.69) | 1.22 (1.00, 1.48) | 0.89 (0.66, 1.20) | 1.27 (0.97, 1.41) | 1.18 (0.85, 1.64) |
| 4 | 1.41 (1.21, 1.64) | 1.11 (0.93, 1.32) | 1.27 (1.07, 1.51) | 0.94 (0.77, 1.15) | 1.27 (1.07, 1.51) | 0.92 (0.75, 1.12) |
| ≥5 | Reference | Reference | Reference | Reference | Reference | Reference |
| Children in Home |  |  |  |  |  |  |
| 1 | 1.94 (1.59, 2.37) | 1.25 (0.95, 1.66) | 1.43 (1.12, 1.83) | 1.14 (0.80, 1.63) | 1.07 (0.86, 1.34) | 0.56 (0.38, 0.83) |
| 2-3 | 1.56 (1.29, 1.88) | 1.14 (0.92, 1.42) | 1.42 (1.14, 1.77) | 1.01 (0.78, 1.30) | 1.32 (1.08, 1.61) | 0.80 (0.63, 1.02) |
| ≥4 | Reference | Reference | Reference | Reference | Reference | Reference |
| Highest Education of Birthing Person |  |  |  |  |  |  |
| <12 years | 0.30 (0.24, 0.37) | 0.40 (0.30, 0.53) | 0.26 (0.20, 0.35) | 0.40 (0.28, 0.56) | 0.13 (0.09, 0.18) | 0.28 (0.19, 0.41) |
| ≥12 years, non-college graduate | 0.40 (0.35, 0.47) | 0.59 (0.49, 0.70) | 0.31 (0.26, 0.37) | 0.54 (0.44, 0.67) | 0.21 (0.18, 0.25) | 0.41 (0.34, 0.50) |
| College graduate | Reference | Reference | Reference | Reference | Reference | Reference |
| Birthing Person Marital Status |  |  |  |  |  |  |
| Married | 2.10 (1.84, 2.38) | 1.47 (1.25, 1.72) | 2.81 (2.40, 3.28) | 1.60 (1.31, 1.95) | 4.40 (3.76, 5.16) | 1.89 (1.54, 2.31) |
| Other Marital Status | Reference | Reference | Reference | Reference | Reference | Reference |
| Language of Interview |  |  |  |  |  |  |
| English | Reference | Reference | Reference | Reference | Reference | Reference |
| Spanish | 1.15 (0.88, 1.50) | 1.76 (1.26, 2.45) | 1.48 (1.07, 2.03) | 2.76 (1.86, 4.11) | 0.75 (0.52, 1.07) | 2.06 (1.31, 3.24) |
| Other | 1.01 (0.61, 1.68) | 1.18 (0.65, 2.13) | 1.13 (0.63, 2.04) | 1.33 (0.68, 2.59) | 0.34 (0.18, 0.64) | 0.40 (0.20, 0.80) |
| Poverty Level, Family Income |  |  |  |  |  |  |
| Below poverty level | 0.42 (0.35, 0.49) | 0.92 (0.74, 1.15) | 0.28 (0.23, 0.34) | 0.68 (0.51, 0.90) | 0.19 (0.15, 0.23) | 0.91 (0.69, 1.19) |
| Above poverty level, ≤$75,000 | 0.61 (0.52, 0.72) | 0.99 (0.83, 1.19) | 0.45 (0.38, 0.54) | 0.87 (0.70, 1.08) | 0.43 (0.36, 0.51) | 1.25 (1.02, 1.53) |
| Above poverty level, >$75,000 | Reference | Reference | Reference | Reference | Reference | Reference |
| Unknown | 0.53 (0.41, 0.69) | 0.86 (0.64, 1.14) | 0.52 (0.38, 0.71) | 0.88 (0.63, 1.24) | 0.35 (0.26, 0.48) | 0.89 (0.63, 1.26) |
| WIC Enrollment |  |  |  |  |  |  |
| Enrolled | 0.44 (0.39, 0.50) | 0.68 (0.57, 0.81) | 0.30 (0.26, 0.35) | 0.48 (0.39, 0.59) | 0.17 (0.14, 0.20) | 0.32 (0.26, 0.39) |
| Not enrolled | Reference | Reference | Reference | Reference | Reference | Reference |
| Unknown or refused to answer | 0.54 (0.27, 1.10) | 0.68 (0.31, 1.46) | 0.51 (0.23, 1.14) | 0.69 (0.29, 1.66) | 0.38 (0.14, 1.04) | 0.58 (0.18, 1.93) |
| ^a^Formula introduced before 6 months or at unknown time, ^b^Formula introduce after 6 completed months or later. PR: prevalence ratio. CI: confidence interval. WIC: Women, Infants and Children. | | | | | | |
